# Antiviral activity of α-helical stapled peptides designed from the HIV-1 capsid dimerization domain

**DOI:** 10.1186/1742-4690-8-28

**Published:** 2011-05-03

**Authors:** Hongtao Zhang, Francesca Curreli, Xihui Zhang, Shibani Bhattacharya, Abdul A Waheed, Alan Cooper, David Cowburn, Eric O Freed, Asim K Debnath

**Affiliations:** 1 Laboratory of Molecular Modeling & Drug Design; Lindsley F. Kimball Research Institute of the New York Blood Center, 310 E 67th Street, New York, NY 10065, USA; 2 New York Structural Biology Center, 89 Convent Avenue, New York, NY, 10027. USA; 3 Virus-Cell Interaction Section, HIV Drug Resistance Program, National Cancer Institute-Frederick, Frederick, MD 21702, USA; 4 School of Chemistry, Joseph Black Building, University of Glasgow, Glasgow G12 8QQ, U.K; 5 Albert Einstein College of Medicine of Yeshiva University, 1300 Morris Park Avenue, Bronx, New York 10461, USA

## Abstract

**Background:**

The C-terminal domain (CTD) of HIV-1 capsid (CA), like full-length CA, forms dimers in solution and CTD dimerization is a major driving force in Gag assembly and maturation. Mutations of the residues at the CTD dimer interface impair virus assembly and render the virus non-infectious. Therefore, the CTD represents a potential target for designing anti-HIV-1 drugs.

**Results:**

Due to the pivotal role of the dimer interface, we reasoned that peptides from the α-helical region of the dimer interface might be effective as decoys to prevent CTD dimer formation. However, these small peptides do not have any structure in solution and they do not penetrate cells. Therefore, we used the hydrocarbon stapling technique to stabilize the α-helical structure and confirmed by confocal microscopy that this modification also made these peptides cell-penetrating. We also confirmed by using isothermal titration calorimetry (ITC), sedimentation equilibrium and NMR that these peptides indeed disrupt dimer formation. In *in vitro *assembly assays, the peptides inhibited mature-like virus particle formation and specifically inhibited HIV-1 production in cell-based assays. These peptides also showed potent antiviral activity against a large panel of laboratory-adapted and primary isolates, including viral strains resistant to inhibitors of reverse transcriptase and protease.

**Conclusions:**

These preliminary data serve as the foundation for designing small, stable, α-helical peptides and small-molecule inhibitors targeted against the CTD dimer interface. The observation that relatively weak CA binders, such as NYAD-201 and NYAD-202, showed specificity and are able to disrupt the CTD dimer is encouraging for further exploration of a much broader class of antiviral compounds targeting CA. We cannot exclude the possibility that the CA-based peptides described here could elicit additional effects on virus replication not directly linked to their ability to bind CA-CTD.

## Background

During HIV-1 assembly and morphogenesis, the structural protein, Gag, organizes into two completely different arrangements, immature and mature forms. In the immature form, Gag remains intact, whereas in the mature form, Gag is cleaved by the viral protease (PR). The formation of this mature particle is essential for HIV-1 infectivity and the capsid protein (CA) obtained from the Gag cleavage product plays a central role in forming the conical virion core that surrounds the viral genome. The CA protein consists of two domains, the N-terminal domain (NTD, aa 1-145) and the C-terminal domain (CTD, aa 151-231). These two domains are connected by a 5-amino-acid linker. The CA-CA contacts in both immature and mature particles have been modeled based on X-ray structures of isolated domains and image reconstructions by cryo-electron microscopy of mature virions and assembled virus-like particles (VLPs) [1-8]. Recently, a pseudo-atomic model of the full-length HIV-1 CA hexameric structure was reported, which provided structural insights on the mechanism of action of some known assembly inhibitors [5,7,9]. HIV-1 CA plays a crucial role in viral assembly, maturation and also early post-entry steps [1-4,6,8]. Mutations in both the NTD and CTD lead to defects in viral assembly and release [10-21]. Taken together, it is evident that CA plays an important role in HIV-1 assembly and maturation, and has been recognized as a potential target for developing a new generation of drugs for AIDS therapy [22-27].

The NTD of CA binds to cyclophilin A [28,29] and is important for viral core formation. However, critical determinants of Gag oligomerization, essential for viral assembly, are located in the CTD [30]. In addition, the CTD encompasses the most conserved segment of Gag known as the major homology region (MHR). Mutation of retroviral CA MHRs leads to severe defects in viral assembly, maturation and infectivity [19,31-37]. The isolated CTD of HIV-1 CA, like full-length CA, forms a dimer in solution. It has been shown that CTD dimerization is a major driving force in Gag assembly and maturation [10,15]. Several structures of the CTD dimer have been reported, providing critical information on the dimer interface [38-41]. Mutation of the interface residues in the CTD monomer disrupts dimer formation [42], impairs CA assembly and abolishes virus infectivity [10,15]. The CTD therefore plays an important role in viral assembly and maturation and is a potential target for developing a new class of anti-HIV-1 drugs [6,43].

Protein-protein interactions play a key role in a range of biological processes such as antigen-antibody interaction [44-46], viral assembly, programmed cell death, cell differentiation and signal transduction. Therefore, controlling these vast arrays of interactions offers opportunities for developing novel therapeutic agents. However, inhibiting these processes by traditional drug discovery techniques may be complicated and challenging due to the shallow binding interfaces and relatively large interfacial areas involved in most protein-protein interactions. Until recently, it was believed to be virtually impossible to inhibit protein-protein interactions to achieve therapeutic benefit. However, this notion is now changing due to recent advances in this area [45-50]. In addition, recent studies on crystallized antigen-antibody complexes have shown that only a limited number of residues from each protein partner are involved in mediating protein-protein interactions. These restricted areas at the binding interfaces are known as 'hot spots', which are small areas of bumps and holes that account for most of the protein interface's free energy of binding. Therefore, it has been established that inhibitors do not have to block the entire binding surface but targeting the 'hot spots' may be sufficient to inhibit protein-protein interactions [51,52].

**Figure 1:**
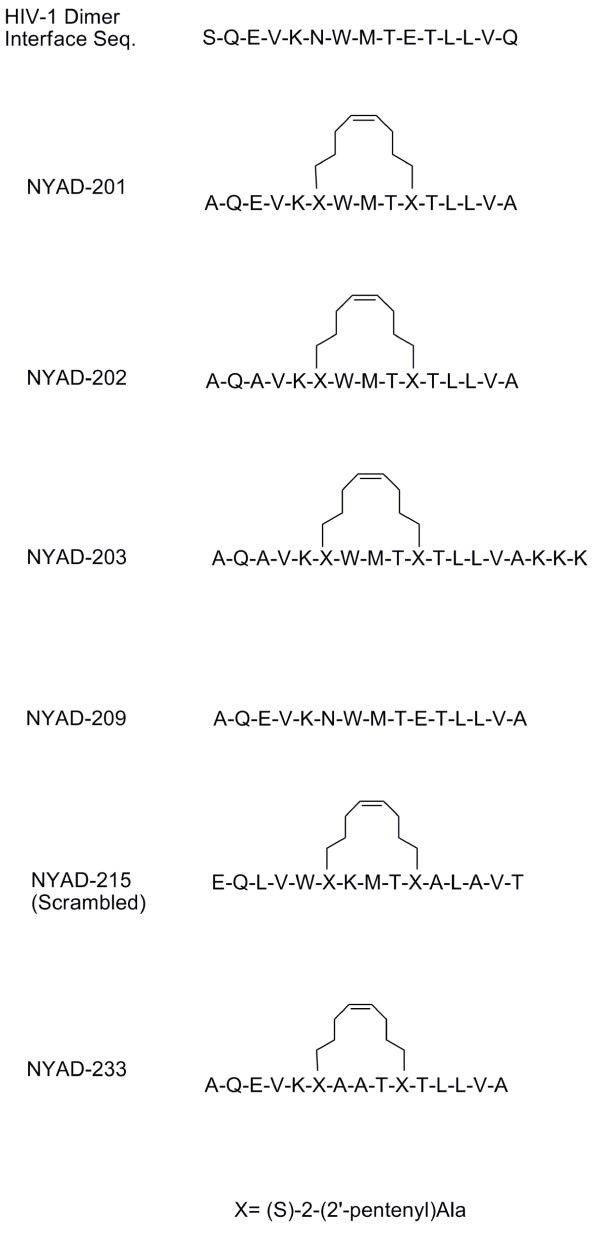
**Sequences and primary structure of linear and hydrocarbon-stapled peptides, indicating the stapling sites**.

Dimeric proteins provide a classical example of protein-protein interactions through surface recognition. There are several examples of competitive inhibitors of protein dimerization that exploit the structure of protein interfaces. For example, interfacial peptides have been shown to inhibit dimerizaton of HIV-1 integrase, protease and reverse transcriptase [53-56]. However, none of these peptides is clinically useful due to their lack of cell permeability. HIV-1 CA forms dimers in solution with modest affinity (K_d _= 18 μM). The dimer interface has been mapped to CTD helix II by various techniques that show significant variability in the packing of the subunit [38,41,57,58]. The solution structure reported by Byeon et al. [57] indicated considerable differences from the structure (2BUO) reported by Ternois et al. [58] and 1A8O reported by Gamble et al. [38] but similar to 1A43 and 1BAJ dimer structures reported by Worthylake et al. [41]. Because the CTD dimer plays a critical role in HIV-1 assembly, we have carefully analyzed the x-ray structure of the CTD dimer (PDB codes: 1a43) and selected a short α-helical segment (aa 178 - 192) from one monomer at the dimer interface region as a starting point for designing inhibitors. These peptides may competitively bind to one monomer of the CTD and prevent CTD dimerization. The biggest challenge facing the use of these short peptides is that they are normally unstructured in solution and do not penetrate cells. Consequently, they cannot be used clinically. We reasoned that hydrocarbon stapling would stabilize the helical structure of the short peptides and would make them cell penetrating as was observed in the case of NYAD-1-type peptides [59] and shown by others [60]. In addition, short hydrocarbon-stapled peptides have been shown to be viable substitutes for small-molecule inhibitors, which can have a larger surface area to bind to the relatively more flat protein binding surface and showed great potential as future drugs [60-63]. We report in this study the rational design of such peptides (Figure 1), which display anti-HIV-1 activity. The data obtained in this study may lay the foundation for the development of small-molecule inhibitors targeted to the CA dimer interface.

## Results

### Stapling enhances the α-helicity of dimer interface peptides

**Figure 2:**
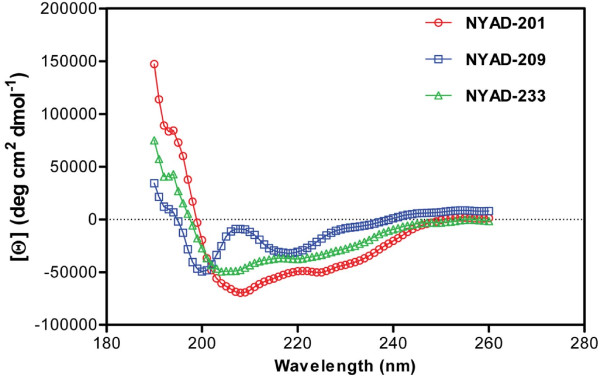
**Circular dichroism (CD) spectroscopy study of NYAD-201, its linear analog NYAD-209 and a double mutant (W184A and M185A) of NYAD-201 (NYAD-233) to determine their secondary structure**. NYAD-201 and NYAD-233 showed characteristic minima at 208 and 222 nm indicating its α-helical structure whereas the linear analog displayed no minima in those regions.

We used circular dichroism spectroscopy to determine the secondary structure characteristics of the stapled dimer interface peptide, NYAD-201, its linear derivative NYAD-209 and a mutant analog of NYAD-201 (NYAD-233). In NYAD-233, the key residues W184 and M185 in NYAD-201 have been replaced with alanine as reported [64]. The stapled peptides NYAD-201 and NYAD-233 showed typical α-helical spectra with minima at 208 and 222 nm. In contrast, the linear peptide NYAD-209 showed two minima, at 200 and 218 nm (Figure 2), suggesting that the secondary structure of the linear peptide adopts neither a random structure nor an α-helical structure. A similar observation with the linear peptide was recently reported [65].

### Stapled peptides penetrate cells

**Figure 3:**
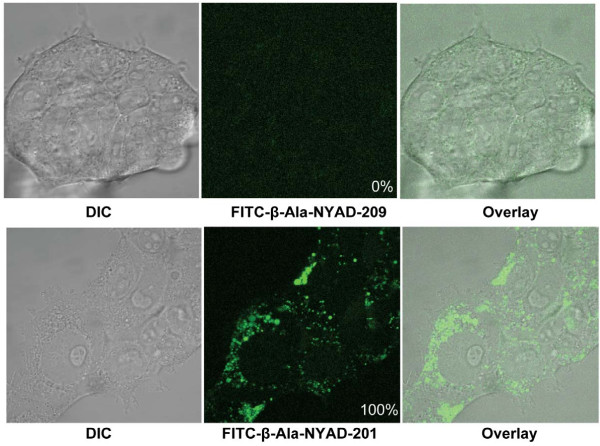
**Cell penetration of NYAD-201 and its linear analog NYAD-209 in 293T cells**. Confocal microscopy images of 293T cells incubated for 20 hours at 37°C with FITC-conjugated peptides. *Upper panel*: *Left*, Differential Interference Contrast (DIC) image of cells with FITC-β-Ala-NYAD-209; *Center*, FITC fluorescent image of the same cells with FITC-β-Ala-NYAD-209; and *Right*, Overlay of DIC and FITC fluorescent images. *Lower panel*: *Left*, DIC image of cells with FITC-β-Ala-NYAD-201; *Center*, FITC fluorescent image of the same cells with FITC-β-Ala-NYAD-201; and *Right*, Overlay of DIC and FITC fluorescent images. A total of 200 cells were scored in each treatment with FITC-β-Ala-NYAD-209 or FITC-β-Ala-NYAD-201. The percentage of cells in the population that exhibited the internal staining is shown at the bottom right of the middle panel.

To show that the stapled peptides NYAD-201 and NYAD-233 penetrate cells, we examined cellular uptake of FITC-conjugated NYAD-201 and NYAD-233 by confocal microscopy. We also used the FITC-conjugated linear peptide NYAD-209 as a control. The data (Figure 3) demonstrate that the linear peptide does not penetrate 293T cells whereas the stapled peptides NYAD-201 and NYAD-233 (Additional file 1, **Figure S1) **penetrate cells.

### Stapled peptides enhance dissociation of dimers

The effect of dimer interface peptides on the dissociation of CA CTD was investigated by two independent biophysical techniques, isothermal titration calorimetry and analytical centrifugation.

**Figure 4:**
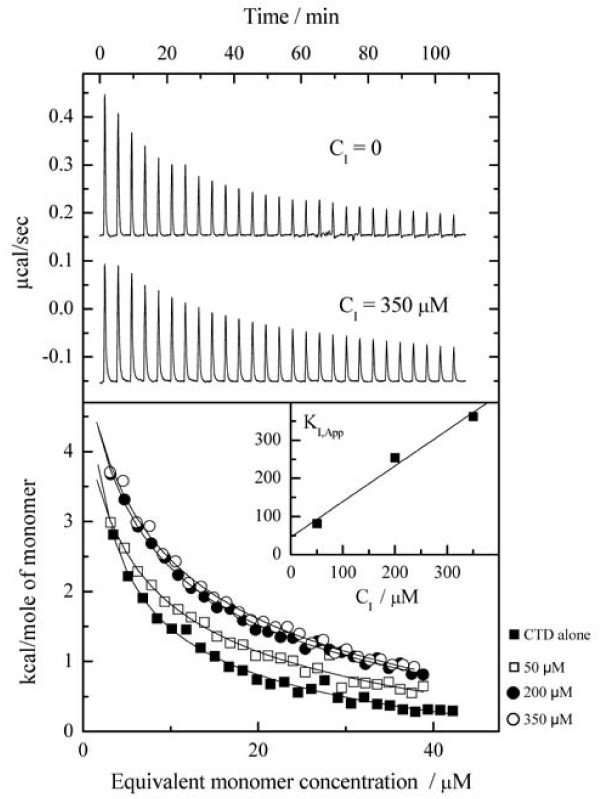
**ITC data for sequential dilution of 10 μl aliquots of CTD (ca. 250 μM) into buffer at 30°;C**. *Upper panel*: raw data comparing endothermic dilution heat effects in the presence and absence of peptide (NYAD-203). *Lower panel*: integrated heats and theoretical fits for dilution of CTD alone (black square) or in the presence of 50 (white square), 200 (black circle), or 350 (white circle) μM peptide inhibitor. The lines are theoretical fits to a dimer dissociation model using parameters listed in Table 1. *Insert*: Concentration dependence of the apparent peptide affinity constant, K_I,App_. Linear extrapolation to zero concentration gives an estimate of K_I _= 45 (±5) μM for binding of peptide to CTD in the absence of peptide self-association.

The effect of the dimer interface peptide, NYAD-203, a soluble analog of NYAD-201, on dimer dissociation was first examined by isothermal titration calorimetry (ITC). Dilution of CTD by sequential ITC injections into buffer, pH 7.3, 30°;C gave a series of endothermic heat pulses (Figure 4) consistent with dissociation of protein oligomers modeled as dimers, with dimer dissociation constant (K_diss_) ~15 μM and dissociation enthalpy (ΔH_diss_) of 10.7 kcal mole^-1^. Similar results were obtained in the presence of stapled peptide, but with progressively increased K_diss _with increasing peptide concentration (Table 1). ITC of an unstapled control peptide (NYAD-401; Seq: IRQGPKEPFRDYVDR) showed no apparent heat effect, supporting the conclusion that binding is significant only for stapled peptides under these conditions. These results are qualitatively consistent with the hypothesis that stapled peptides bind competitively to the CTD monomer and inhibit dimerization. For such a mechanism, the apparent dimer dissociation constant should depend on the free peptide (inhibitor) concentration ([I]) and binding affinity (K_I_) as follows:

**Table 1:** Apparent thermodynamic parameters for dissociation of CTD dimers, as determined by ITC dilution experiments at 30°;C in the presence of stapled peptide NYAD-203 with concentration, C_I_

C_I_/μM	K_diss_/μM	ΔH_diss_/kcal mole^-1^	200 μM CTD (f_m_)*
0	15.3 (±3.6)	10.7 (±0.7)	18%

50	31.2 (±6.6)	9.1 (±0.3)	24%

200	33.2 (±3.9)	10.8 (±0.2)	25%

350	40.5 (±6.2)	10.9 (±0.2)	27%

* f_m _is the estimated fraction of CTD occurring as monomers in solution under these conditions, calculated from the monomer-dimer equilibrium expressions.

or an approximate form that might be valid at high inhibitor concentrations:

where C_i _is the total peptide inhibitor concentration in the mixture. This analysis is, however, further complicated by apparent self-association of the peptide (see NMR section) that reduces the availability of free monomer (lower [I]) at higher concentrations and thus leads to underestimation of peptide binding affinities and a concentration dependence of apparent K_i_. However, by use of the above equations and extrapolation to low concentrations, we obtain a value of K_i _= 40 (±5) μM for the binding of stapled peptide (NYAD-203) monomer to CTD under these conditions. This value of K_i _is slightly higher, though of comparable order of magnitude to the self-association dimerization constant of the CTD, thus validating the primary hypothesis that the subunit interaction free energy is dominated by this peptide region.

The impact of NYAD-203 on the dissociation of the CA dimer was also evaluated by analytical ultracentrifugation. Combined sedimentation velocity and equilibrium approaches were used to characterize dimerization of CA.

In the first set of experiments, the CA alone and the CA with different ratios of NYAD-203 were analyzed by sedimentation velocity centrifugation. The CA alone (30 μΜ) yielded a single symmetrical peak (data not shown). CA (30 μΜ) with different ratios of NYAD-203 (CA: NYAD-203 = 1:1 or 1:3) also yielded a single symmetrical peak (data not shown).

**Figure 5:**
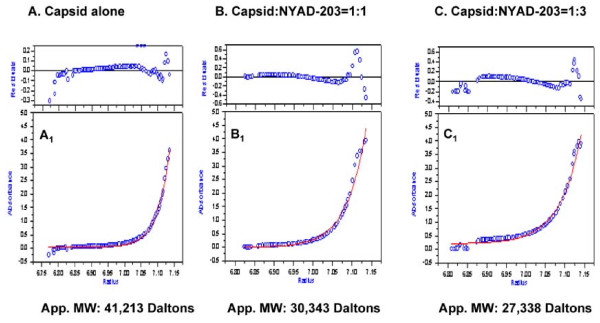
**Sedimentation equilibrium analysis of NYAD-203 on CA**. Data were collected at 20,000 rpm, 25°C and pH 7.3 using the Beckman XL-A/XL-I Analytical ultracentrifugation. The CA concentration was fixed at 30 μM. **(A, B & C) **The residual difference between the fitted curve and the experimental data and (**A_1_, B_1 _& C_1_) **Plot of Absorbance at 280 nm vs. centrifugal radius in the absence (control) or presence of 30 μM and 90 μM of NYAD-203, respectively. The open circles represent the experimental data and the solid lines are the best fit using an ideal single-species model.

In the second set of experiments, the possible effect of NYAD-203 on dimer stability was analyzed by sedimentation equilibrium. As shown in Figure 5, the apparent molecular weight for CA in the absence of or in the presence of different ratios of NYAD-203 was determined. The CA alone (30 μΜ) yielded an apparent molecular weight of 41,213 Daltons. CA: NYAD-203 at a 1:1 molar ratio yielded an apparent molecular weight of 30,343 Daltons and at a 1:3 ratio yielded an apparent molecular weight of 27,338 Daltons.

Theoretically, the observed decrease in the apparent molecular weight could be due to the establishment of rapidly reversible monomer-dimer dissociation equilibrium. Therefore, the data point to a shift in the association-dissociation equilibrium, indicative of an increased dissociation to monomer in the presence of higher doses of NYAD-203.

### Mapping interactions between CTD and stapled peptides

**Figure 6:**
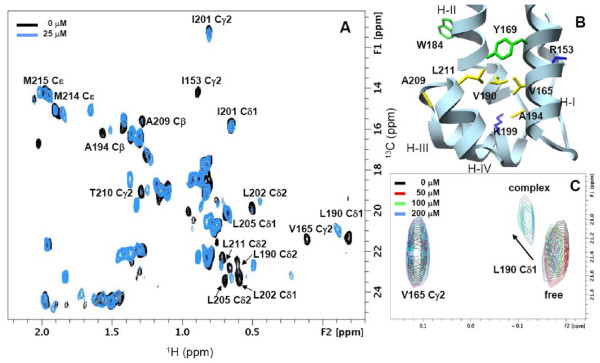
**The ^1^H-^13^C HSQC spectra of CTD complexed with the peptide NYAD-203**. (A) The methyl region of the free and peptide (25 μM) complexed [U C^13^, N^15^] CTD from the **^1^H-^13^C **HSQC spectra is shown in the panel. (B) CTD residues perturbed upon binding to the peptide and could be assigned unambiguously are annotated (bold/black) in the structure. (C) The population weighted changes in the Cδ2 peak intensity of Leu190 from CTD in the free and bound states at four peptide concentrations.

At very low CTD concentrations (<10 μM) the protein is predominantly monomeric (>90%) and upon binding to the peptide, the structural perturbations induce chemical shift changes in the aliphatic resonances followed by ^1^H-^13^C HSQC spectra shown in Figure 6. A characteristic feature of the CTD dimer interface in solution is the overall variability of Helix II, a source for significant exchange broadening of resonances from residues located in Helix II at the dimer interface [66]. Although the selective loss of NMR signals from Helix II limited the information available for mapping the exact binding site of the peptide we observed concentration-dependent chemical shift changes elsewhere in the hydrophobic core of the protein (Figure 6B). The extent of chemical shift perturbation strongly suggests a reorganization of the helical core structure of the CTD upon complex formation with the peptide. In Figure 6C, the resonances from Leu190 Cδ1/Hδ1 and Lys199 Cα/Hα (Additional file 2, **Figure S2**), are each represented by two cross-peaks instead of a single peak in the ^1^H-^13^C HSQC spectra. The differential intensities of the two peaks are population weighted corresponding to the free and bound states, respectively. Similar population-weighted cross-peaks in the 'slow exchange regime' of the NMR time scale were observed through the titration elsewhere in the protein. As expected, upon increasing the peptide concentration to 25 μM the cross-peak intensity of Leu190 Cδ2 in the bound fraction of CTD increases to 100% (Figure 6A), but surprisingly diminishes at higher peptide concentrations (Figure 6C). Structural characterization of the isolated peptide NYAD-203 by NMR revealed a helical structure for residues 3-14 that self-associates to form polymeric species (**Figure S2**). The four-fold excess of peptide required to saturate 7 μM CTD yields a binding affinity less than 10 μM. The affinity of NYAD-203 for monomeric CTD at low concentrations is apparently higher compared to the value of 40 μM obtained from the ITC experiments. It is very likely that at elevated peptide and protein concentrations used in those experiments self-association dominates and to a degree competes with the interaction between the peptide and protein.

### NYAD-201 specifically inhibits HIV-1 production

**Figure 7:**
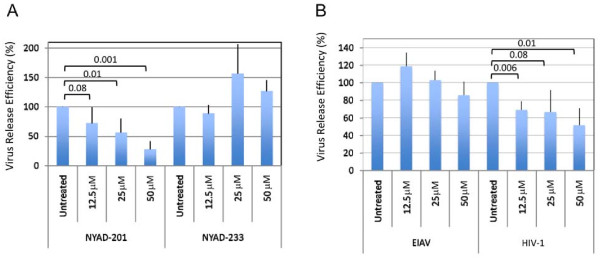
**NYAD-201 inhibits HIV-1 but not EIAV particle production**. (A) 293T cells were transfected with pNL4-3 and 5 h after transfection treated with indicated concentrations of NYAD-201 or NYAD-233 for 18 h. Cells were then metabolically labeled with [^35^S]Met/Cys for 2 h. Cells were lysed and virions were collected by ultracentrifugation. Cell and virus lysates were immunoprecipitated with HIV-Ig and subjected to SDS-PAGE; protein bands were quantified by phosphorimager analysis. HIV-1 release efficiency was calculated as the amount of virion-associated p24 relative to total (cell + virion) Gag. (B) 293T cells were transfected with CMV-driven vectors expressing EIAV (pPRE-Gag) or HIV-1 (pCMVdeltaR8.2/PR-) Gag and treated with NYAD-201 at indicated concentrations. After 18 h, peptide-treated cells were metabolically labeled with [^35^S]Met/Cys for 2 h (HIV-1) or 5 h (EIAV) and immunoprecipitated with HIV-Ig or anti-EIAV antiserum. The release efficiency was calculated as the amount of virion-associated Pr55^Gag ^to total Pr55^Gag ^in cells and virions. P values were calculated by Student's t-test, with P < 0.01 considered significant. N = 3, ± SD.

To investigate whether NYAD-201 disrupts HIV-1 assembly in cell culture, we transfected 293T cells with the full-length HIV-1 molecular clone pNL4-3, and 5 h after transfection treated with varying concentrations of NYAD-201 for 18-20 h. Cells were then metabolically labeled for 2 h with [^35^S]Met-Cys, and labeled viral proteins in cell and virion lysates were immunoprecipitated with HIV-Ig and analyzed by SDS-PAGE followed by fluorography [67]. As shown in Figure 7A, the release efficiency of HIV-1 was reduced in a concentration-dependent manner; with 50 μM NYAD-201 there was a 3-fold reduction in virus release. As a control, we also included peptide NYAD-233, in which the key dimer interface residues "WM" in NYAD-201 were changed to Ala-Ala (Figure 1). No defect in virus release efficiency was observed with the NYAD-233 control peptide (Figure 7A). To examine whether the inhibition of virus release mediated by NYAD-201 is specific to HIV-1, we tested the effect of this peptide on the release of another lentivirus, equine infectious anemia virus (EIAV) in 293T cells. Interestingly, the release of EIAV particles was not impaired by NYAD-201 (Figure 7B), indicating that the inhibiting effect of NYAD-201 on HIV-1 particle production was not the result of non-specific effects such as cytotoxicity. To address the possibility that the difference in sensitivity of HIV-1 vs. EIAV to NYAD-201 was the result of Gag being processed by PR (for HIV-1 in Figure 7A) or not being processed (for EIAV in Figure 7B), we tested in parallel a CMV promoter-driven HIV-1 Gag expression vector. Again, we observed that HIV-1 VLP production was modestly reduced whereas EIAV particle production was not affected (Figure 7B). Together, these results suggest that binding of NYAD-201 to the CA CTD modestly but specifically interferes with HIV-1 particle production in cells.

### Stapled peptides inhibit mature-like particle formation

To determine whether NYAD-201 disrupts the assembly of both immature or mature-like particles we set up two *in vitro *assembly assays [59]. We used full-length Gag proteins to form spherical immature-like particles. The effect of inhibitors on immature particle assembly was studied by performing assembly reactions in the presence of varying doses of NYAD-201.

**Figure 8:**
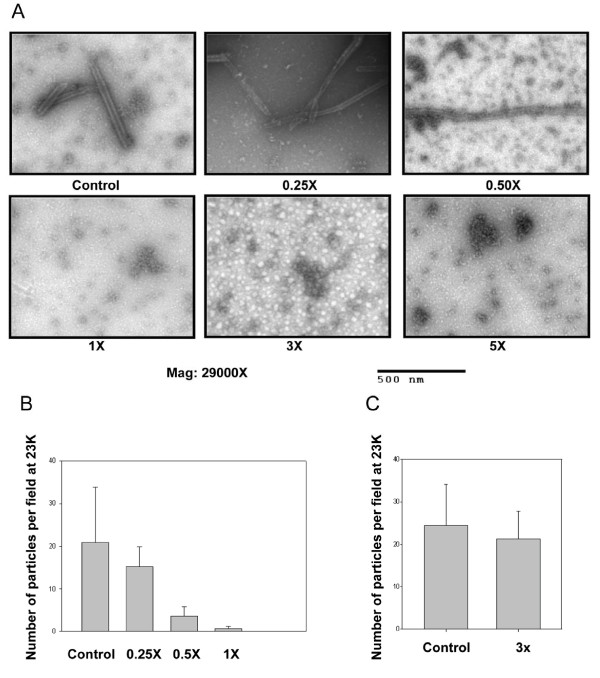
**Inhibition of *in vitro *assembly by NYAD-201**. (A) Negatively stained EM images of mature-like particles resulting from *in vitro *assembly of CA proteins in the presence of no peptide (Control), 0.25-, 0.5-, 1.0-, 3.0- and 5-fold molar equivalent of NYAD-201. (B) A dose-response effect of NYAD-201 on the tube-like mature particle formation. (C) *In vitro *assembly of CA proteins in the presence of no peptide (control) and 3-fold molar equivalent of NYAD-233.

NYAD-201 failed to disrupt immature-like particle formation even at molar equivalent dose (data not shown). For the mature-like particles, we obtained tube-shaped particles from purified CA, [3,68] which when exposed to NYAD-201, showed a clear dose-response effect (Figure 8A and 8B). At even a 0.25-fold molar equivalent of NYAD-201, substantial disruption of tube-shaped particles was observed. Complete disruption was attained at molar equivalent and higher doses (Figure 8A and 8B). NYAD-233, the mutant stapled peptide lacking the key "WM" motif (Figure 1), failed to disrupt mature-like particles even at 3-fold molar equivalent dose (Figure 8C). A scrambled hydrocarbon-stapled peptide, NYAD-215, showed no effect on the formation of mature-like particles (data not shown). The rationale behind using CA instead of CANC to form the mature-like particles was to confirm that NYAD-201 targets CA only [59].

### Stapled dimer interface peptides target infectivity at a post-entry stage

We used human SupT1 cells, incubated with GFP-Vpr-labeled HIV-1 virions in the presence of NYAD-201 or the peptide-based HIV-1 fusion inhibitor T-20 (enfuvirtide), as a control to assess virus entry (data not shown). T-20 is the first clinically used peptide-based drug designed to inhibit HIV-1 entry [69-72]. Fluorescence-activated cell sorting (FACS) analysis demonstrated that 36%, 41% and 38% of SupT1 cells were GFP+ at 50 μM, 25 μM or 12.5 μM NYAD-201, respectively, similar to the levels observed (35%) in the absence of NYAD-201. In contrast, T-20 at 111 nM substantially inhibited viral entry. The ability of T-20 to significantly reduce the GFP+ signal strongly argues that the signal is derived from particles that have entered the cell rather than from those that remain bound to the cell surface.

**Figure 9:**
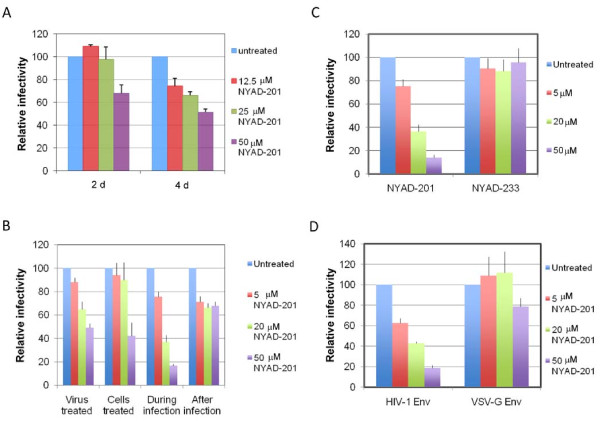
**NYAD-201 inhibits HIV-1 infectivity**. (A) Five hours post transfection with pNL4-3, 293T cells were treated with 12.5, 25, and 50 μM NYAD-201 for 2 or 4 days and supernatant were collected and monitored for RT activity. Approximately 7 μl RT-normalized virus stocks were used to infect the TZM-bl cells for 2 h in a total volume of 200 μl. This approach led to a dilution of peptide concentration from the producer cell supernatant of ~30-fold. Two days after infection, cells were washed, lysed and assayed for luciferase activity. (B) Cells or virions were treated with 5, 20 or 50 μM NYAD-201 before, during, or after infection as described in Methods. Infection of TZM-bl cells was carried out for 2 h and two days after infection cells were washed, lysed and assayed for luciferase activity. (C) NYAD-201 or NYAD-233 were added to TZM-bl cells at indicated concentrations during the 2 h infection. Two days after infection cells were washed and luciferase activity was measured as in A. (D) HIV-1 virions were pseudotyped with VSV-G by cotransfecting the Env-defective pNL4-3 derivative (pNL4-3/KFS) with the VSV-G expression vector pHCMV-G. RT-normalized WT and VSV-G pseudotyped virions were used to infect TZM-bl cells in the presence of the indicated concentrations of NYAD-201 for 2 h. Two days after infection, luciferase activity was measured in cell lysates. N = 4 for A, B, D; n = 3 for C; ± SD.

Since NYAD-201 is a CA-binding peptide and its interaction with the CTD inhibits viral core formation, we tested whether NYAD-201 could inhibit virion infectivity. To this end, we performed single-cycle infectivity assays in the TZM-bl indicator cell line [73,74]. The assays were conducted under several different conditions (Figure 9). Virus-producing cells were incubated with 12.5, 25 and 50 μM NYAD-201 for two or four days and the released virions were collected. RT-normalized virus stocks were used to infect TZM-bl cells. As shown in Figure 9A, virions produced from cells treated for four days with NYAD-201 showed a two-fold reduction in infectivity. We note that peptide from the producer cell supernatant was diluted ~30-fold in the TZM-bl infectivity assay (see Figure 9 legend). Furthermore, the concentration of active peptide in the medium after two or four days in culture is likely to be considerably lower than the concentration added initially. We also treated virions with NYAD-201 prior to infection, treated the target cells before or after infection, or treated with peptide during the two-hour infection period. Interestingly, treating the virions or target cells prior to infection reduced the infectivity by two- to three-fold; however, if the peptide was present during the two-hour infection period the inhibition was more severe (5-6 fold). Treating cells postinfection imposed a modest (two-fold) defect in virion infectivity (Figure 9B). As a control for these infectivity assays, we used the control peptide NYAD-233 (Figure 1). Again, peptide NYAD-201 inhibited virus infectivity by >5-fold when present during the two-hour infection period, whereas NYAD-233 had no effect on particle infectivity (Figure 9C).

These results suggest that in addition to inhibiting virion assembly and maturation, NYAD-201 may also affect an entry and/or early post-entry step in the replication cycle. To investigate this issue in more detail, we tested whether pseudotyping virions with VSV-G could bypass the infectivity block. TZM-bl cells were infected with VSV-G-pseudotyped NL4-3 in the presence of NYAD-201. Intriguingly, the infectivity of VSV-G-pseudotyped HIV-1 was not inhibited under these conditions (Figure 9D). This Env-dependent effect suggests that the infectivity defect imposed by NYAD-201 can be reversed by altering the virus entry pathway. It is important to note that there is precedent in the literature for a CA-dependent defect in core assembly being reversible by VSV-G pseudotyping [75].

### Anti-HIV-1 activity and cytotoxicity of stapled peptides in cell-based assays

We measured the anti-HIV-1 activity of NYAD-201 and its analogs (Figure 1) in a cell-based assay using several laboratory-adapted and primary isolates in MT-2 cells and PBMC, respectively. The inhibition of p24 production in MT-2 cells was measured over a range of concentrations and the concentration required to inhibit 50% (IC_50_) of the p24 production was calculated. The results in Table 2 indicate that NYAD-201 and its analogs efficiently inhibited a broad range of HIV-1 strains, representing different subtypes, which use R5, X4 or R5X4 coreceptors including one X4-tropic RT-inhibitor-resistant (AZT-R) strain and one X4-tropic PR-inhibitor-resistant strain. The stapled peptides inhibited the laboratory strains with low μM potency (IC_50 _~ 3-6 μM), and both R5- and X4-tropic viruses were inhibited with similar potency. We also tested the linear peptide and two control hydrocarbon-stapled peptides, NYAD-215 and NYAD-233. Neither of these peptides showed any antiviral activity even at a 100 μΜ dose (data not shown).

**Table 2:** Antiviral activity (IC_50_) and cytotoxicity (CC_50_) of NYAD-201, NYAD-202 and NYAD-203 in laboratory-adapted and primary HIV-1 isolates

	HIV-1 virus	Subtype	Cell Type	Coreceptor	IC_50 _(μM±SD)		
					NYAD-201	NYAD-202	NYAD-203
**Laboratory Strains**	IIIB	B	MT-2	X4	4.29 ± 0.62	2.36 ± 0.33	6.29 ± 0.54
	MN	B	MT-2	X4	3.03 ± 0.61	2.47 ± 0.71	
	SF2	B	MT-2	R5X4	5.06 ± 1.37	4.48 ± 0.84	
	RF	B	MT-2	X4	2.84 ± 0.63	2.64 ± 0.39	
	BaL	B	PBMC	R5	4.73 ± 1.92	2.23 ± 0.44	
	89.6	B	PBMC	R5X4	5.21 ± 0.87	3.47 ± 0.22	

**RT-Resistant Isolate**	AZT-R	B	MT-2	X4	8.0 ± 1.27	4.53 ± 1.19	11.1 ± 3.82

**PR-Resistant Isolate**	**HIV-1_RF/L-323-12-3_**	B	MT-2	X4	5.6 ± 0.5	3.5 ± 0.6	

**Primary isolates**	93RW024	A	PBMC	R5X4	9.88 ± 0.3	3.71 ± 0.19	
	92UG029	A	PBMC	X4	7.88 ± 1.01	3.97 ± 0.47	
	92US657	B	PBMC	R5	6.72 ± 0.98	3.61 ± 0.57	
	93IN101	C	PBMC	R5	1.58 ± 0.57	5.53 ± 0.39	
	98CN009	C	PBMC	R5	5.31 ± 0.83	4.08 ± 0.92	
	CMU02	EA	PBMC	X4	7.36 ± 0.69	4.2 ± 0.01	
	93BR020	F	PBMC	R5X4	2.78 ± 0.57	2.51 ± 0.43	
	RU570	G	PBMC	R5	7.48 ± 1.21	6.34 ± 2.22	
	BCF02	(Group 0)	PBMC	R5	15.84 ± 3.43	6.2 ± 1.2	

	**Peptides**	**CC_50 _(μM) in MT-2**			**CC_50 _(μM)in PBMC**		

	**NYAD-201**	>115			>115		

	**NYAD-202**	30.2 ± 4.32			>116		

	**NYAD-203**	13.24 ± 0.5			15.96 ± 1.47		

We also tested the inhibition of NYAD-201 and its analogs against a panel of primary HIV-1 isolates in PBMC representing mostly group M (subtypes from A to G) with diverse coreceptor usage. The peptides showed inhibition against all primary isolates tested including one from group O. These peptides showed similar inhibitory activities against this diverse range of primary isolates, except against group O strain, which showed somewhat reduced inhibition, indicating its effectiveness against a wide range of HIV-1 isolates.

The cytotoxicity of the stapled peptides was assessed by the XTT method in both MT-2 cells and PBMC. Cytotoxicity assays were performed in parallel with the HIV-1 inhibition assays. The CC_50 _(concentration of inhibitor required to produce 50% cytotoxicity) values of NYAD-201 in MT-2 cells and PBMC were >115 μM. NYAD-202 is more cytotoxic in MT-2 cells (30) than in PBMC (>116 μM). However, NYAD-203 was cytotoxic, as observed previously for NYAD-13 [59].

## Discussion

In this study, we describe the rational design of peptide-based inhibitors derived from the HIV-1 CA dimerization domain. This approach is based on the hypothesis that these peptides will act as decoys and bind to the monomeric CA, thereby preventing CTD dimer formation, a critical step in virus assembly and maturation. We chose a fifteen residue linear segment from the dimer interface (aa 178-192) to design the decoy based on the HIV-1 CA dimer structure as well as the biophysical studies of a dimer interface peptide, CAC1, which was shown to form a heterocomplex with CA CTD with an apparent dissociation constant of 50 μM [65]. However, it is well-known that peptides of such short length tend to exist as random structures despite the fact that the secondary and tertiary structures of this segment in the CTD protein are α-helical. Since the α-helical structure is critical for dimer formation we used a hydrocarbon stapling technique [60,61,76,77] to stabilize the α-helical structure of this short peptide. We selected residues 183 (*i*) and 187 (*i+4*) of the α-helical segment for stapling because they are centrally located opposite from the dimer interface and expected not to interfere with the binding of this modified stapled peptide to the CTD monomer. We further modified other residues at the N- and C-termini based on the thermodynamic dissection analysis of the dimer interface [78] which showed that mutation of certain residues enhanced the association constant. It appears that the CTD dimer is required to have weak association due to other critical functions where monomers may play an important role; however, to design effective inhibitors from the dimer interface peptide we need to redesign the short peptides with enhanced binding affinity towards the CTD monomer. Based on this requirement, we synthesized NYAD-201, NYAD-202 and NYAD-203, a soluble analog of NYAD-201. We also synthesized the linear analog of NYAD-201 (NYAD-209) and a mutant analog of NYAD-201 (NYAD-233) after mutating two key dimer-interface residues, W184A and M185A. CD analysis confirmed that NYAD-201 and the mutant stapled peptide, NYAD-233, had the characteristic helical spectrum, whereas the linear analog, NYAD-209, showed no α-helicity. Since assembly and formation of virus particles are intracellular events, these peptides must penetrate cells to exert their action by preventing dimer formation and thereby inhibiting particle production. Although the mechanism of cell penetration is not clearly understood, these stapled peptides penetrated cells as reported for similar stapled peptides [60,61,79]. The modified peptides, except the mutant peptide NYAD-233, inhibited particle formation *in vitro*. These results support the structural model that a relaxed dimer can produce immature particles; however, it is critical to achieve a compact CTD configuration to form mature particles [80]. This finding indicates that peptide-based inhibitors targeted to different sites on the CTD, as described in this study as well as in our earlier report [59], may provide mechanistic insights into the viral assembly and maturation process and may help in elucidating the structural requirements in forming immature and mature virus particles.

We observe that NYAD-201 has a modest effect on HIV-1 particle production. The finding that this peptide does not inhibit EIAV VLP production and that the control peptide NYAD-233 has no effect on either HIV-1 or EIAV particle production indicates that the effect is specific to the HIV-1 CA-CTD dimer interface. However, at this time, the mechanism of action of NYAD-201 with respect to VLP formation is not clear. The reduction in particle production could be due to effects on a variety of aspects of Gag function (e.g., Gag stability, trafficking, etc.) rather than to impaired immature assembly *per se*.

We utilized ITC and sedimentation equilibrium centrifugation to measure the effect of these stapled peptides on the dissociation of the CTD dimer. The enhanced dissociation of the CTD dimer in the presence of progressively increasing concentrations of stapled peptide was an indirect measure of the inhibitory activity by ITC. Despite the fact that NYAD-203, the soluble analog of NYAD-201, fit a dimer dissociation model, the effect on dissociation was modest. These data are in contrast to the potent inhibition by these peptides in antiviral assays. Similar discrepancies have been noted with CAP-1, a CA-NTD-targeted compound [81]. A likely explanation for these results is that the local concentration of Gag is much higher (14 mM) [81] within the densely packed lattice of the mature particle thus ensuring that the bound peptide saturates a fraction of the available sites. Hence, the relatively low affinity of the peptide does not diminish its activity towards the mature virus particle to the same extent that we observe a limited effect on dissociation of the CTD in solution.

A characteristic feature of the CTD dimer interface in solution is the overall flexibility of Helix II proposed to facilitate rearrangement of the Gag molecules through various stages of mature particle assembly [82]. Previous studies have established that mutant CTD can exist as a stable monomer and is remarkably similar to the subunits of the native dimer structure [64]. The stability of the monomer structure is then not tightly coupled with dimerization and it can exist as an independent domain without unfolding completely [83,84]. The monomeric CTD mutant (W184A/M185A) has a hydrophobic pocket on the distal side of the dimer interface known to bind peptides [85] but in this instance has no measurable affinity for NYAD-203 (data not shown). Therefore we hypothesize that the stapled peptide NYAD-203, a structural mimic of helix II, interacts with the transiently exposed helix-II at the interface of the dimer by a dynamic mechanism. The peptide is unlikely to favor a single binding mode and the evidence for dynamic exchange between conformational states is seen through the excessive broadening and/or presence of multiple complex peaks through the titration by NMR. Preliminary analysis of the NMR data suggests that the CTD structure complexed with the peptide undergoes a conformational change in the core packing of hydrophobic residues. Despite the modest peptide affinity, the partial loss of the CTD dimer interface and subtle changes in the monomer structure of the Gag molecules is expected to disrupt and inhibit the assembly of the capsid particle. Contrary to the biophysical and NMR evidence in support of direct interactions between the peptide and CTD, *in vitro *the homologous peptide NYAD-201 appears to have no effect on the immature particles but only disrupts the mature particles. A possible explanation for this observation can be traced to the closely-packed lattice of cup shaped NTD hexamers in the immature virus particle stabilized from the bottom by SP1 interactions reinforced by inter- and intra-hexamer CTD contacts [80,86]. Following the proteolysis of the CTD-SP1 junction the fullerene-like structure of the capsid is assembled from NTD hexamers augmented by CTD dimerization across NTD rings [5]. In the mature particle the spacing of the CTD domains in each CA hexamer is increased and the concomitant dimer contacts are only possible between two capsids. We speculate that this reorganization of the subunits of CA is an important structural determinant that facilitates greater access of CTD to the peptide inhibitor in the lattice of the mature particle thus increasing its effectiveness. Unfortunately the current resolution of the reconstructed CTD models lacks the atomic detail necessary to make definitive comparisons between the dynamics and buried surface triggered by the viral capsid assembly mechanism.

These peptides showed potential as antivirals against several laboratory-adapted and primary HIV-1 isolates, including one RT-inhibitor-resistant and one PR-inhibitor-resistant strain. NYAD-201 and NYAD-202 showed encouragingly broad-spectrum activity, irrespective of the subtype, coreceptor use and drug-resistance status of the isolates. However, NYAD-203 was cytotoxic, as was NYAD-13 [59], a similarly prepared soluble analog of NYAD-1. Despite the fact that we have yet to confirm the mechanism of action of these peptides, we have demonstrated specificity by using a peptide (NYAD-233) mutated in the two critical amino acids (W184 and M185) at the dimer interface. This control dimer-interface-disrupted peptide showed a loss of activity in virus particle production and infectivity assays.

## Conclusions

In conclusion, these preliminary data serve as the foundation for designing small, stable, α-helical peptides and small-molecule inhibitors targeted to the CTD dimer interface. The observation that relatively weak CA binders, such as NYAD-201 and NYAD-202, are sufficient to dissociate and deform the virion cores offers encouragement for the exploration of a much broader class of antiviral compounds targeting CA. We cannot exclude the possibility that the CA-based peptides described here could elicit additional effects on virus replication not directly linked to their ability to bind the CA-CTD. For example, the observation that under some circumstances the effect of NYAD-201 on virus infectivity could be mitigated by VSV-G pseudotyping suggests that this peptide may also impose an entry-related defect; however, these peptides failed to inhibit virion uptake. Furthermore, there is precedent for CA-related defects being reversible by VSV-G pseudotyping. Brun et al. [75] reported that a mutation in the linker domain between CA-NTD and CA-CTD disrupts core stability. This core disruption imposed an infectivity defect that was reversed in virions bearing VSV-G, instead of HIV-1 Env [75]. Similarly, a mutation in the CA NTD (N74D) has been shown to bypass the need for the nuclear import factor transportin-SR2 in HIV-1 infection [87]; however, the transportin-SR2 independence conferred by the N74D mutation was Env glycoprotein dependent in that VSV-G-pseudotyped N74D virus could infect cells depleted of transportin-SR2 but N74D virus bearing HIV-1 Env could not [88]. These observations may be attributable to differences in the uncoating process associated with different routes of viral entry mediated by VSV-G and HIV-1 Env. By analogy, the inhibitory peptides reported here could disrupt CA post-entry function in a manner reversible by a VSV-G-mediated entry route. Further studies will be required to more fully understand the role that CA plays in early post-entry events and to more precisely define the step at which inhibitory peptides like NYAD-201 act.

## Methods

### Reagents

AZT (Cat# 3485), T-20, [fusion inhibitor from Roche (Cat# 9845)], MT-2 cells (Dr. D. Richman), Sup-T1 cells (Dr. James Hoxie), laboratory adapted and primary HIV-1 strains, U87-T4-CXCR4 cells were obtained through the NIH AIDS Research and Reference Reagent Program. 293T cells were obtained from the American Type Culture Collection. PBMC were processed from blood obtained from the New York Blood Center.

### Molecular cloning, protein expression and purification

pET14b or pET28a plasmids encode Gag-derived proteins from the HIV-1_NL4-3 _strain. The full-length gag expression vector was obtained from the NIH AIDS Research and Reference Reagent Program. The CA coding region was obtained by PCR amplification and was inserted into the pET28a vector. The C-CA DNA fragment was provided by Drs Ming Luo and Peter E Prevelige Jr. The C-CA DNA fragment was subcloned into the pET14b vector. The C-CA mutant (W184A/M185A) was generated from pET14b-C-CA by overlapping PCR. The corresponding proteins (including ^15^N or ^15^N/^13^C labeled mutant C-CA and C-CA) were expressed and purified as described previously^10, 47, 53^. Protein concentrations were determined with the A_280 _molar extinction coefficients of 2.980 M^-1^cm^-1^(C-CA, mutant), 8,480 M^-1^cm^-1^(C-CA), 33,460 M^-1^cm^-1^(CA) and 64,400 M^-1^cm^-1^(Gag), respectively.

### Virus assembly and release assays and immunoprecipitation analysis

For metabolic radiolabeling assays, 293T cells (plated at 2.5 × 10^5 ^cells/well in 12-well dishes) were transfected with the HIV-1 molecular clone pNL4-3 [89], the EIAV Gag expression construct pPRE-Gag [90,91], or the HIV-1 Gag expression vector pCMVdeltaR8.2/PR-. This clone was constructed from pCMVdelR8.2 [92]by introducing the PR-inactivating mutation from pNL4-3/PR- [93]. Five h posttransfection, cells were treated with the indicated concentrations of NYAD-201 or control peptide NYAD-233 for 16-20 h. One day posttransfection, cells were metabolically labeled for 2 h (HIV-1) or 5 h (EIAV) with 250 μCi [^35^S]Met/Cys. The labeled virions were pelleted in an ultracentrifuge and cell and virus lysates were immunoprecipitated and subjected to SDS-PAGE. Quantification was performed by Phosphorimager analysis, and virus release efficiency was calculated as the amount of virion-associated Gag as a fraction of total (cell- plus virion-associated) Gag synthesized during the metabolic labeling period. HIV-1 immunoglobulin (HIV-Ig) antiserum was obtained from the NIH AIDS Research and Reference Reagent Program. EIAV horse serum was kindly provided by Dr. Ronald Montelaro (University of Pittsburgh).

### Synthesis of stapled peptides

The peptides were synthesized manually by Fmoc solid phase synthesis as described previously [59].

### CD spectroscopy

CD spectra were obtained on a Jasco J-715 Spectropolarimeter (Jasco Inc, Japan) at 20°C using the standard measurement parameters in Tris-HCl buffer (20 mM Tris, pH 8.0) in the presence of 1-15% (vol/vol) acetonitrile at a final concentration of 125-500 μM. In all the samples, the final concentrations of peptides and salt were always the same, and the spectra were corrected by subtracting the CD spectra of the appropriate reference solvent.

### Confocal microscopy

For the cell penetration study, 293T cells were seeded in 4-well chamber plates and incubated at 37°C with 5 μΜ of FITC-conjugated peptides in serum-free medium for 4 h and/or an additional 16 h in medium containing serum. After three washes with 1× PBS, live cells were examined and imaged under a Zeiss LSM510 laser scanning confocal microscope (Zeiss).

### Electron microscopy to study inhibition of *in vitro *assembly by peptides

*In vitro *assembly systems were set up as described [15,68,94,95] with minor modification. We used 50 mM Na_2_HPO_4_, pH 8.0 as the dialysis buffer. The buffer used for assembly studies also contained 0.1~2 M of NaCl. 500-Da-MWCO dialysis tubes (Spectra/Por) were used for the dialysis of peptides. Briefly, stock proteins were adjusted to the appropriate concentration (25 μΜ for Gag proteins or 50 μΜ for CA proteins) with the Na_2_HPO_4 _buffer at pH 8.0. After addition of 5% total *E. coli *RNA (RNA:protein = 1:20 by weight), incubation with varied doses of NYAD-201 and NYAD-233 for 30 min at 4°C, the samples were dialyzed overnight in Na_2_HPO_4 _buffer at pH 8.0 containing 100 mM NaCl at 4°C. For assembly of CA mature-like particles, addition of 5% total *E. coli *RNA was omitted. Negative staining was used to check the assembly. Carbon-coated copper grids (200 mesh size; EM Sciences) were treated with 20 μl of poly-L-lysine (1 mg/ml; Sigma) for 2 min. 20 μl of reaction solution was placed onto the grid for 2 min. Spotted grids were then stained with 30 μl of uranyl acetate solution for 2 min. Excess stain was removed, and grids were air-dried. Specimens were examined with a TECNAI G^2 ^electron microscope.

### ITC Analysis

The influence of the peptides on dimer dissociation of wild type CTD (wtCTD) was investigated by ITC (MicroCal VP-ITC) following established protocols [96,97]. Proteins were exhaustively dialyzed against the 25 mM sodium phosphate buffer (pH 7.3) prior to experimental measurements. In a typical experiment, the ITC injection syringe was loaded with 250 μM protein, dissolved in dialysis buffer or dialysis buffer/peptide mixture and the calorimetric cell (ca. 1.4 ml active volume) initially contained only the identical buffer mixture. Typically titrations consisted of 28 injections of 10 μl, with 240-s equilibration between injections. The thermodynamic parameters were obtained and data were analyzed using MicroCal Origin 7.0 software, using an updated and corrected (June 2008) version of the dissociation analysis procedure, validated by comparison with earlier analysis methods [96,97].

### Sedimentation Velocity Centrifugation

Sedimentation velocity experiments were performed using a ProteomeLab XL-A analytical ultracentrifuge (Beckman Coulter) with 2-channel centerpieces in an An-60Ti rotor at 42,000 rpm and 25°C. Radial scans at a single wavelength (typically 280 nm) were taken at 300 s intervals. The solvent density and viscosity and the protein partial specific volume were calculated using the program SEDNTERP. Protein samples were dialyzed into a buffer containing 50 mM sodium phosphate buffer (pH 7.3). The data were fitted to the continuous size-distribution functions g(*S*) using the program SEDFIT.

### Sedimentation Equilibrium Centrifugation

Sedimentation equilibrium experiments were performed using a ProteomeLab XL-A analytical ultracentrifuge using 6-channel centerpieces in an An-60Ti rotor at 25°C. Protein samples were dialyzed into 50 mM sodium phosphate buffer (pH 7.3). Samples were centrifuged for ~16 h at 28000 rpm, 32000 rpm or 36000 rpm until they reached equilibrium and no further change was seen in the distribution. Radial scans were measured at 280 nm. The data were fitted to an ideal single-species model as well as a rapid monomer-dimer equilibrium using the Beckman XL-A/XL-I Data Analysis Software (Version 6.03).

### NMR Experiments

Standard ^1^H-^13^C HSQC spectra were acquired on a 7 μM sample of [U-^13^C,^15^N] CTD complexed with NYAD-203 at various concentrations ranging from 0-500 μM prepared in 20 mM phosphate buffer (90% H_2_O/10% D_2_O) at pH 6.5. The protein concentration was constant in every sample. The titration was followed at 25°C by acquiring data on a 900 MHz *AVANCE *II spectrometer equipped with TCI CryoProbe using 128 transients for signal averaging. The NMR data were processed and analyzed in Topspin 2.1.

### Preparation of GFP-Vpr-labeled HIV-1 virions

Green fluorescent protein (GFP)-expressing virions were produced by cotransfection of 293T cells (plated in a T75 flask) with HIV-1 pNL4-3 proviral DNA (15 μg) and an expression vector (15 μg) encoding a GFP-Vpr fusion protein. After 48 h, the virus-containing supernatant was subjected first to low-speed centrifugation to remove cells and debris and then to ultracentrifugation at 20,000 rpm in an SW41 rotor for 2 h at 4°C to sediment viral particles. The virus-containing pellet was resuspended in complete medium (0.5 ml) and stored in aliquots at -70°C. Twenty thousand SupT1 cells were incubated with ~0.3 μg of p24-normalized virus particles in the absence or presence of different dosages of NYAD-201 and T-20 for 3 hours. After a treatment with 1× Trypsin-EDTA (GIBCO) and one wash with 1× PBS, the cells were fixed and subjected to FACS analysis after another washing with 1× PBS.

### Single-cycle infectivity assay

The single-cycle infectivity assays were performed by using the TZM-bl indicator cell line (obtained from John Kappes through the NIAID AIDS Reagent Program), which contains integrated copies of the β-galactosidase and luciferase genes under control of the HIV-1 LTR [73,98]. One day before infection, TZM-bl cells (4 × 10^4 ^cells/well) were seeded in a 24-well tissue culture plate. RT-normalized (200,000 RT cpm) virus stocks were used to infect the TZM-bl cells. NYAD-201 was added to cells before or after infection or was present during the 2 h infection period as indicated. To generate VSV-G-pseudotyped viruses, 293T cells were cotransfected with the Env-defective pNL4-3 derivative (pNL4-3/KFS) [99] and the VSV-G expression vector pHCMV-G [100]. After 48 h, cells were washed with PBS and lysed in luciferase lysis buffer (Promega) and infection efficiency was determined by measuring luciferase activity. The samples were assayed with the Promega luciferase assay substrate (Promega) in a multifunctional microplate reader.

For experiments in which the producer cells were treated with NYAD-201 for 2 or 4 days, 293T cells were transfected with pNL4-3. Five hour posttransfection, cells were washed and medium containing the indicated concentrations of NYAD-201 was added. Virus supernatant was collected 2 or 4 days posttransfection and RT-normalized virus was used for infectivity assays.

*In vitro *experiments were performed as follows: 293T cells in 60-mm dishes were transfected as above and the virus supernatant was collected 24 h posttransfection. Virus supernatants from several 60-mm dishes were pooled and aliquoted into several tubes. NYAD-201 was added to the virus supernatants at indicated concentrations and incubated for approximately 20 h at 37°C. A small aliquot of virus supernatant was stored for infectivity assays.

### Measurement of multi-round HIV-1 replication

The inhibitory activity of NYAD-201 and other stapled peptides on replication by laboratory-adapted HIV-1 strains was determined as previously described [101] with minor modification. In brief, 1 × 10^4 ^MT-2 cells were infected with HIV-1 at 100 TCID_50 _(50% tissue culture infective dose) (0.01MOI) in 200 μl RPMI 1640 medium containing 10% FBS in the presence or absence of peptides at graded concentrations overnight. The culture supernatants were then removed and fresh media containing freshly prepared test peptide were added. On the fourth day post-infection, 100 μl of culture supernatants were collected from each well, mixed with equal volume of 5% Triton X-100 and tested for p24 antigen by ELISA.

The inhibitory activity of peptides on replication by primary HIV-1 isolates was determined as previously described [102]. PBMCs were isolated from the blood of healthy donors at the New York Blood Center by standard density gradient centrifugation using Histopaque-1077 (Sigma-Aldrich). The cells were cultured at 37°;C for 2 h. Nonadherent cells were collected and resuspended at 5 × 10^6 ^cells/ml RPMI-1640 medium containing 10% FBS, 5 μg/ml PHA, and 100 U/ml IL-2 (Sigma-Aldrich), followed by incubation at 37°;C for 3 days. The PHA-stimulated cells (5 × 10^4 ^cells/ml) were infected with primary HIV-1 isolates at 500 TCID_50 _(0.01 MOI) in the absence or presence of peptide inhibitor at graded concentrations. Culture media were changed every 3 days and replaced with fresh media containing freshly prepared inhibitor. The supernatants were collected 7 days post-infection and tested for p24 antigen by ELISA. The percent inhibition of p24 production, IC_50 _and IC_90 _values were calculated by the GraphPad Prism software (GraphPad Software Inc.).

### Cytotoxicity assay

Cytotoxicity of peptides in MT-2 cells and PBMC was measured by the XTT [(sodium 3'-(1-(phenylamino)-carbonyl)-3,4-tetrazolium-bis(4-methoxy-6-nitro) bezenesulfonic acid hydrate)] method as previously described [102]. Briefly, for MT-2 cells, 100 μl of a peptide at graded concentrations was added to an equal volume of cells (1 × 10^5 ^cells/ml) in 96-well plates followed by incubation at 37°C for 4 days, which ran parallel to the neutralization assay in MT-2 (except medium was added instead of virus). In the case of PBMC, 5 × 10^5 ^cells/ml were used and the cytotoxicity was measured after 7 days. After addition of XTT (PolySciences, Inc.), the soluble intracellular formazan was quantitated colorimetrically at 450 nm 4 h later with a reference at 620 nm. The percent of cytotoxicity and the CC_50 _values were calculated as above.

## Competing interests

The authors declare that they have no competing interest

## Authors' contributions

HZ carried out in vitro and cell-based assays, cell penetration study, circular dichroism, electron microscopy, isothermal titration calorimetry and sedimentation equilibrium analyses and analyzed data; FC carried out the antiviral assays and analyzed data; XZ helped in preparing and purifying proteins; SB performed all NMR related studies, analyzed the data and written part of the manuscript; AAW performed the study on HIV and EIAV assembly and release, prepared the corresponding figures; AC, analyzed the ITC data and written part of the manuscript; DC, analyzed the NMR data and written part of the manuscript; EOF analyzed the data and written and edited the manuscript, AKD conceived the study, designed all stapled and other peptides, planned experiments, analyzed data, written and edited the manuscript. All authors read and approved the final manuscript.

## Supplementary Material

Additional file 1 **Fig-S1. Cell penetration of NYAD-233 and its linear analog NYAD-209 in 293T cells**. Confocal microscopy images of 293T cells incubated for 20 hours at 37°C with FITC-conjugated peptides. *Upper panel*: *Left*, Differential Interference Contrast (DIC) image of cells with FITC-β-Ala-NYAD-209; *Center*, FITC fluorescent image of the same cells with FITC-β-Ala-NYAD-209; and *Right*, Overlay of DIC and FITC fluorescent images. *Lower panel*: *Left*, DIC image of cells with FITC-β-Ala-NYAD-233; *Center*, FITC fluorescent image of the same cells with FITC-β-Ala-NYAD-233; and *Right*, Overlay of DIC and FITC fluorescent images. A total of 200 cells were scored in each treatment with FITC-β-Ala-NYAD-209 or FITC-β-Ala-NYAD-233. The percentage of cells in the population that exhibited the internal staining is shown at the bottom right of the middle panel. Click here for file

Additional file 2 **Fig-S2. The ^1^H-^13^C HSQC spectra of CTD complexed with the peptide NYAD-203**. The three panels display the effect of the titration on select crosspeaks from the aliphatic region through the titration. Resonances from the protein that could be assigned unambiguously are annotated in black and those from the excess peptide are indicated in blue. (A) The population weighted changes in the Lys199 Cα cross-peak intensity in the free and bound states at four peptide concentrations. (B) and (C) The effect of peptide addition on W184 Cα and Cδ1 cross-peak. (D) Standard one dimension proton NMR spectra of NYAD-203 at various concentrations in 20 mM phosphate buffer at pH 7.0 and 288 K. The NMR data were processed and analyzed in Topspin 2.1. Click here for file
