# Supplementary figures and images for: Antiviral activity of α-helical stapled peptides designed from the HIV-1 capsid dimerization domain
Source: Retrovirology. 2011 May 3;8:28. doi: 10.1186/1742-4690-8-28 (PMC3097154; doi:10.1186/1742-4690-8-28)

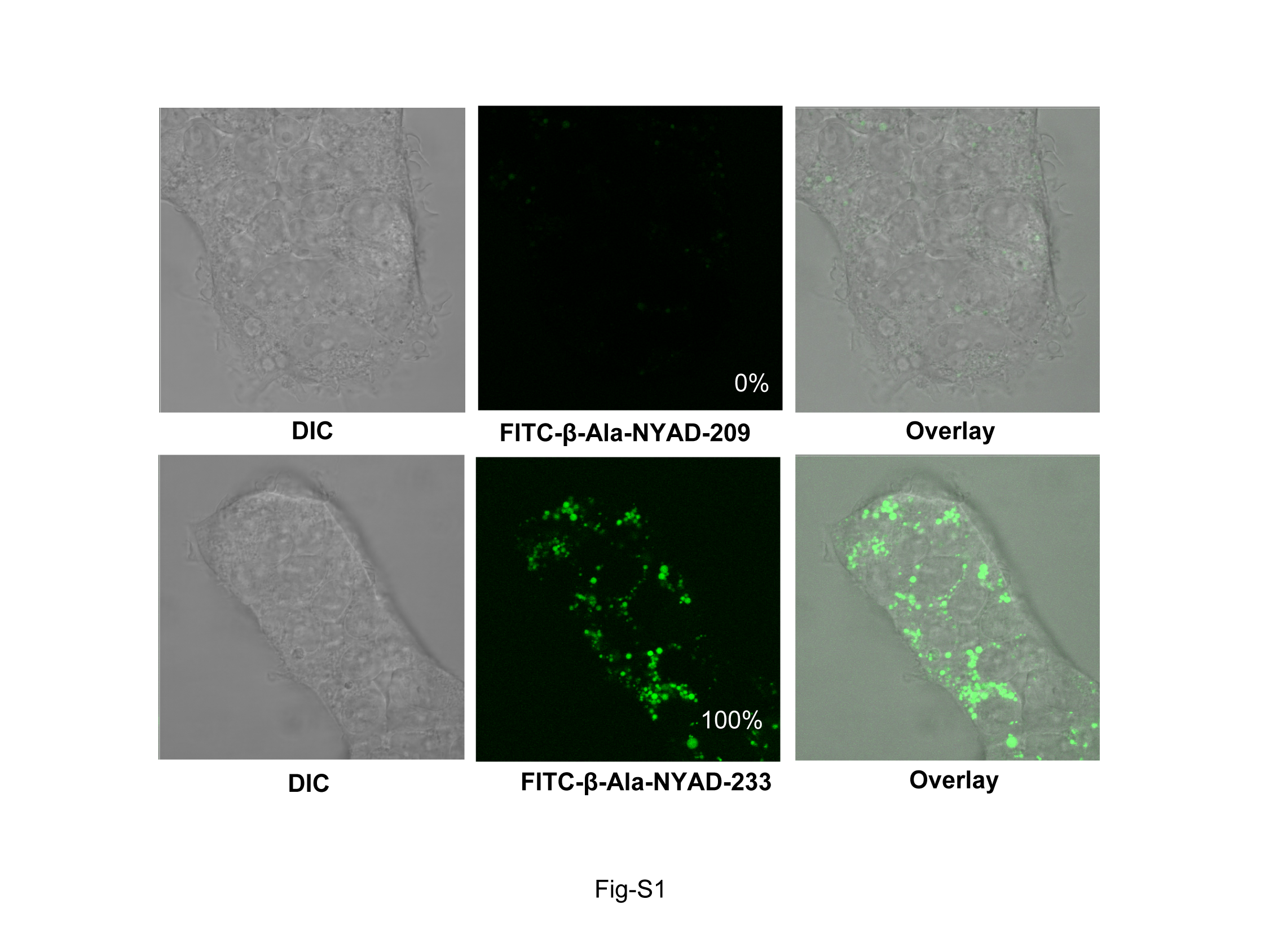

Supplement: Additional file 1 — Fig-S1. Cell penetration of NYAD-233 and its linear analog NYAD-209 in 293T cells. Confocal microscopy images of 293T cells incubated for 20 hours at 37°C with FITC-conjugated peptides. Upper panel: Left, Differential Interference Contrast (DIC) image of cells with FITC-β-Ala-NYAD-209; Center, FITC fluorescent image of the same cells with FITC-β-Ala-NYAD-209; and Right, Overlay of DIC and FITC fluorescent images. Lower panel: Left, DIC image of cells with FITC-β-Ala-NYAD-233; Center, FITC fluorescent image of the same cells with FITC-β-Ala-NYAD-233; and Right, Overlay of DIC and FITC fluorescent images. A total of 200 cells were scored in each treatment with FITC-β-Ala-NYAD-209 or FITC-β-Ala-NYAD-233. The percentage of cells in the population that exhibited the internal staining is shown at the bottom right of the middle panel. [file 1742-4690-8-28-S1.TIFF]

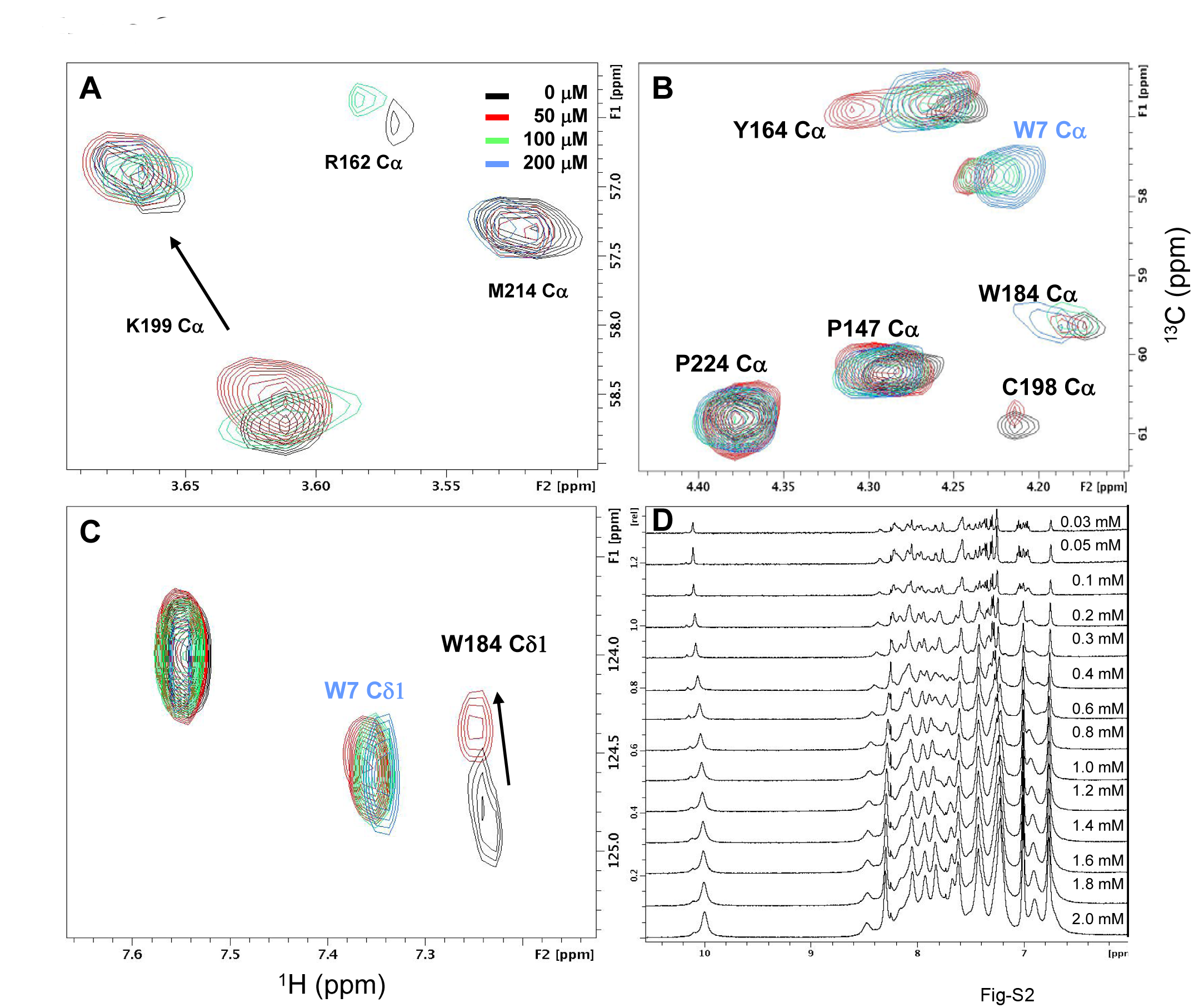

Supplement: Additional file 2 — Fig-S2. The 1H-13C HSQC spectra of CTD complexed with the peptide NYAD-203. The three panels display the effect of the titration on select crosspeaks from the aliphatic region through the titration. Resonances from the protein that could be assigned unambiguously are annotated in black and those from the excess peptide are indicated in blue. (A) The population weighted changes in the Lys199 Cα cross-peak intensity in the free and bound states at four peptide concentrations. (B) and (C) The effect of peptide addition on W184 Cα and Cδ1 cross-peak. (D) Standard one dimension proton NMR spectra of NYAD-203 at various concentrations in 20 mM phosphate buffer at pH 7.0 and 288 K. The NMR data were processed and analyzed in Topspin 2.1. [file 1742-4690-8-28-S2.TIFF]
